# Green spaces, blue spaces and human health: an updated umbrella review of epidemiological meta-analyses

**DOI:** 10.3389/fpubh.2025.1505292

**Published:** 2025-05-22

**Authors:** XiaoWen Wang, Bowen Feng, Juan Wang

**Affiliations:** ^1^College of Geography and Planning, Chengdu University of Technology, Chengdu, China; ^2^Qilu Hospital, Shandong University, Jinan, China

**Keywords:** green spaces, blue spaces, human health, human settlements, umbrella review

## Abstract

**Introduction:**

Green spaces have been recognized for their ecological services, including air purification and biodiversity protection, which contribute to the enhancement of life quality and well-being. However, despite the surge in research evaluating the impact of blue-green spaces on health outcomes, we still lack a definitive understanding of how green and blue spaces affect human health outcomes. To assess the impact of blue-green spaces on human health outcomes, we systematically summarized and evaluated the relationship between green and blue spaces and human health through an umbrella review of epidemiological meta-analyses up to the year 2024.

**Methods:**

The study follows the PRISMA guidelines and includes meta analyses from PubMed, Embase, and Cochrane databases, focusing on evidence and methodological improvements. Inclusion criteria encompass studies on human populations, exposure to green and blue spaces, and health outcomes such as mortality, disease risk, and physiological indicators. Data extraction and quality assessment of evidence and methods are conducted using the GRADE system and AMSTAR 2 tool.

**Results:**

We find that green space exposure is associated with reduced all-cause mortality, mortality from cardiovascular diseases, incidence of diabetes and metabolic syndrome, low birth weight, and mental health improvements. Blue spaces also show positive associations with beneficial health outcomes, including reduced obesity rates and improved psychological well-being. However, the evidence regarding green space exposure and specific health outcomes such as cancer, asthma, and allergic rhinitis remains heterogeneous and unclear.

**Discussion:**

Green and blue spaces clearly have some impact on health. For some outcomes, the effects are robust. This article emphasizes the importance of improving residents’ health through urban planning in public health strategies.

**Systematic review registration:**

https://www.crd.york.ac.uk/PROSPERO/, identifier [CRD42024533346].

## Graphical abstract

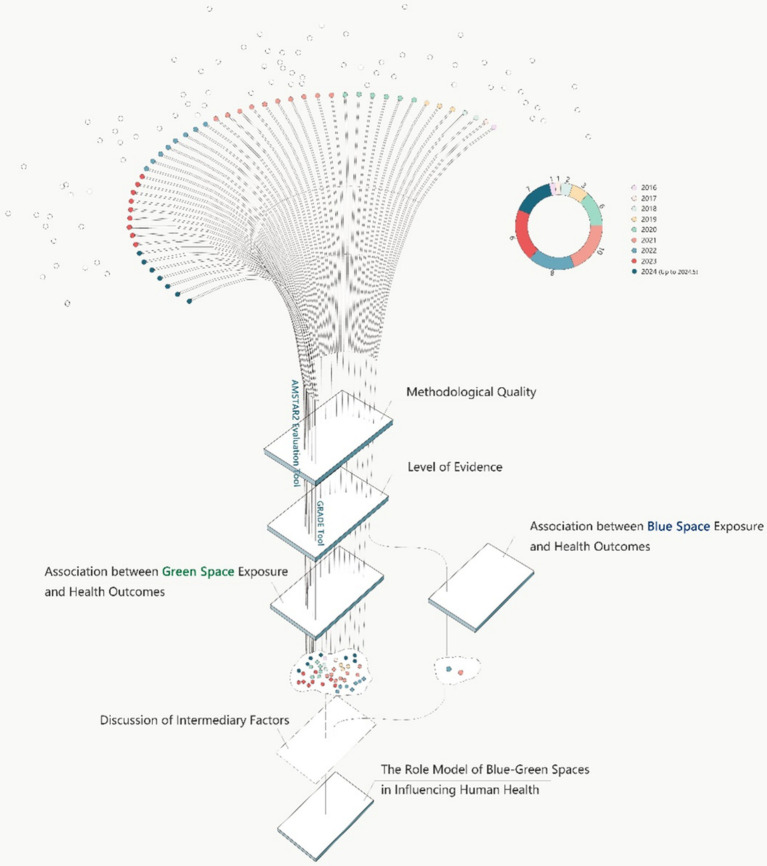

## 1 Introduction

As an essential component of the human living ecosystem, green spaces have increasingly drawn attention for their impact on human health (1). Green spaces not only provide recreational areas but also offer various ecological services such as air purification, mitigation of the heat island effect, and biodiversity protection (2). These services play a crucial role in enhancing residents’ quality of life and physical and mental well-being. Numerous studies in recent years have confirmed the positive association between green spaces and human health, including promoting physical activity (3), reducing mortality rates (4), lowering the risk of cardiovascular diseases (5), and improving mental health (6).

Although a systematic review of this field was conducted in 2021 (7), significant advancements in the study of the relationship between green spaces and human health have been made over the past 3 years due to continuous improvements in research methodologies and the emergence of new scientific evidence. These advancements include identifying and confirming more health outcomes associated with green spaces, such as the risks of type 2 diabetes (8, 9) and obesity (101, 102). Correspondingly, new literature reviews and meta-analyses have increased not only in quantity but also in the depth and breadth of research. These studies cover various aspects, from the impact of green spaces on specific health outcomes to how green space characteristics (10), frequency of exposure (11), and socioeconomic factors modulate this relationship (12). There are also articles where no relationship is found or a negative relationship in some results. Some studies have even presented contradictory results. We are drowning in a sea of evidence, yet we still lack a definitive understanding of the impact of green spaces on human health outcomes.

This study aims to systematically summarize and evaluate all meta-analyses on the relationship between green spaces and human health up to 2024. 5 through an umbrella review of evidence provided by epidemiological studies (13). Compared to the 2021 study, we focus on new evidence, improvements in research methods, potential differences, and controversies to supplement and update the existing knowledge system. We conduct relatively more rigorous semi-quantitative analyses using advanced methodological tools (e.g., AMSTAR2, GRADE) to obtain updated and more reliable evidence. Additionally, we will update the assessment of another important system in the living environment: the impact of blue spaces on human health, specifically, it refers to the living environment and water-related environment, such as lakes, rivers, wetlands, coasts, and other water bodies (14). Like green spaces, blue spaces provide opportunities for recreation and relaxation, and emerging research suggests that they can also have significant effects on mental health, physical well-being, and social interactions (15). We aim to provide the latest scientific evidence for public health decision-makers, urban planners, and environmental protection policymakers, guiding them in formulating more effective strategies to promote the protection and utilization of green spaces, thereby improving the health and quality of life of urban residents, and to anticipate future research directions.

## 2 Methods

We conducted a systematic umbrella review of the meta-analyses following the Preferred Reporting Items for Systematic Reviews and Meta-Analyses (PRISMA) guidelines (16) (Supplementary Table S1). The protocol for this umbrella review has been registered in the International Prospective Register of Systematic Reviews (Prospero), ID: CRD42024533346.

### 2.1 Inclusion criteria and searches

We systematically searched three international electronic databases: PubMed, Embase, and Cochrane. Our search strategy used terms related to green spaces (“urban forest,” “green area,” “open space,” “greenness,” “greenspace,” “greenery,” “urban park,” “green infrastructure,” “urban vegetation,” “green land,” and greenspace and land type indicators: “normalized difference vegetation index, (NDVI),” “Soil Adjusted Vegetation Index,” “Enhanced Vegetation Index,” and “Leaf area index”), blue spaces, gardening, forest bathing, and exposure to natural environments, as well as systematic reviews and meta-analyses (“systematic review” or “meta-analysis”) (Supplementary Table S2). The data included studies published up to May 30, 2024. We restricted our search to research articles. We manually cross-checked the results of the title and abstract searches to remove duplicates and extended the search to papers and reports cited in the literature but not in the above databases.

Two researchers (W.X. and F.B.) independently screened the titles and abstracts to determine study inclusion. Discrepancies were resolved through discussion with a third author (W.J.). Our inclusion criteria were as follows: (1) Population—studies on human populations regardless of age, gender, race, geographic region, and health status; (2) Exposure—studies on exposure to green and blue spaces, including residential green spaces (assessed using vegetation indices, proportion of green space, proximity to green spaces, or the amount of green space in a specific area), activities conducted in natural environments (e.g., exercising in nature, gardening) and exposure to blue spaces; (3) Comparison—studies comparing health impacts of different levels of green space exposure; (4) Outcomes—studies investigating any health outcomes, such as mortality, disease risk, prevalence, incidence, and physiological indicators. We applied no specific design limitations to the primary studies under consideration. However, we deliberately excluded studies not written in English, not involving human subjects, and conference abstracts from our review.

### 2.2 Data extraction

Two authors (W.X. and F.B.) independently extracted the data, with discrepancies resolved through discussion with a third author (W.J.). For every systematic review that met eligibility criteria, we extracted key details, including the authorship, the year of publication, the type of study design—be it observational or interventional—the principal findings, and the defining traits of the encompassed primary studies. These characteristics included age range, sample size, methods of assessing green/blue spaces, health outcomes, effect sizes, 95% confidence intervals, and statistical significance.

### 2.3 Credibility and quality assessment of evidence and methods

The quality of included meta-analyses was evaluated by using AMSTAR 2 (A Measurement Tool to Assess Systematic Reviews—second edition) (17). Two authors independently assessed each item of the tool, and any discrepancies were discussed with a third author. According to AMSTAR2 checklist, items 2, 4, 7, 9, 11, 13, and 15 were identified as critical domains, as a basis for evaluate the characteristics of systematic reviews included in the umbrella review (Supplementary Table S3).

We used the GRADE (Grading of Recommendations, Assessment, Development, and Evaluation) system to assess the quality of evidence for each outcome in each meta-analysis, categorizing them as “high,” “moderate,” “low,” or “very low” (18). According to GRADE standards, all observational studies are considered low-quality evidence. The GRADE method includes eight criteria, five of which can lower confidence in the accuracy of effect estimates, resulting in downgrading: risk of bias, inconsistency of results, indirectness of evidence, imprecision, and publication bias. Additionally, three criteria can increase or enhance confidence: a large magnitude of effect with no plausible confounders, a dose–response gradient, and a study where all plausible residual confounders would reduce the effect or suggest a spurious effect if not controlled. Two authors independently assessed each item based on the content of the articles. Heterogeneity was primarily evaluated using the I^2^ value: we defined 0–30% as low, 30–70% as moderate, and above 70% as high heterogeneity. If high heterogeneity was observed, the evidence score was downgraded.

### 2.4 Data synthesis

**Figure 1:**
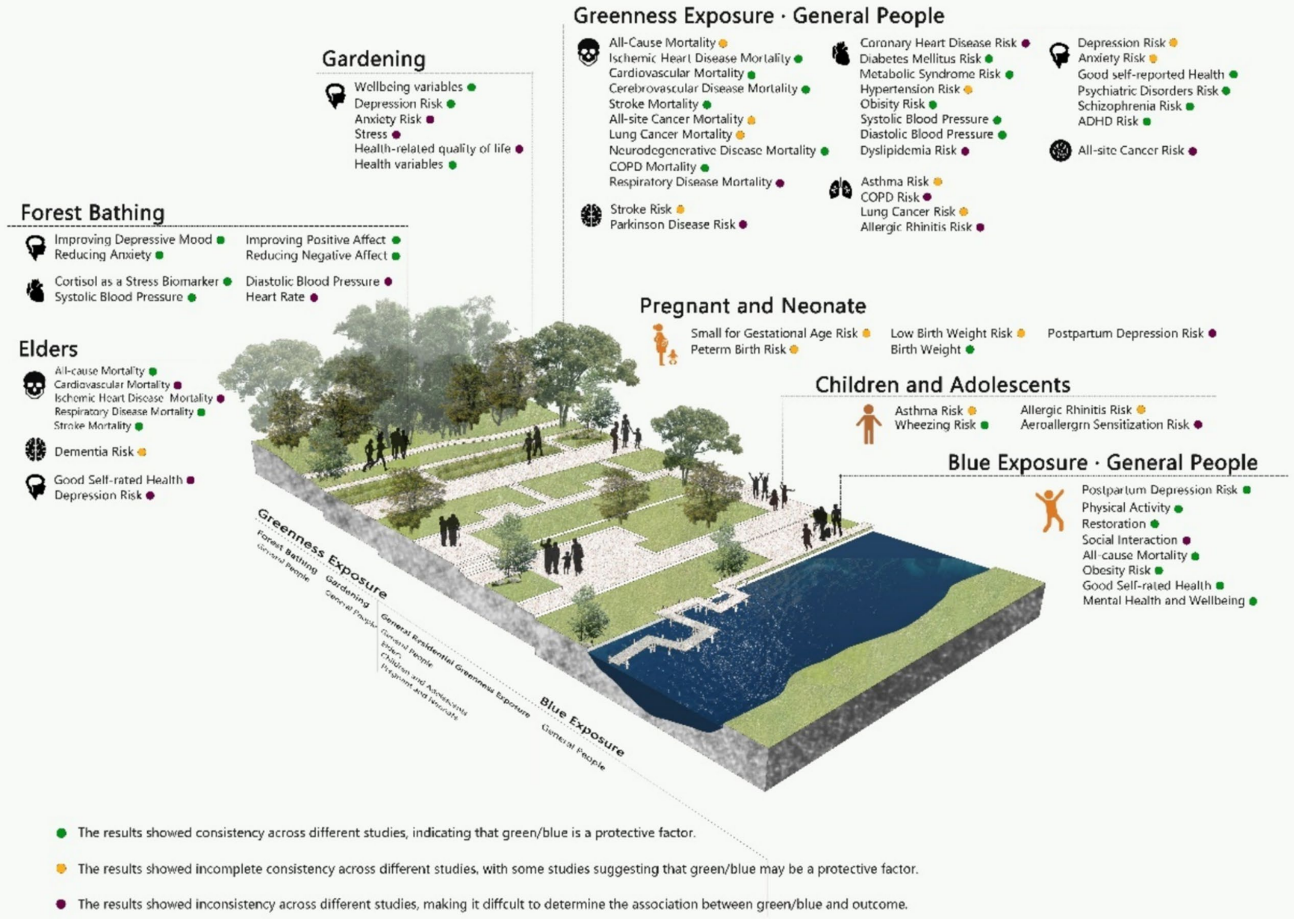
The association between green/blue spaces and human health outcomes. COPD, Chronic Obstructive Pulmonary Disease; ADHD, Attention Deficit Hyperactivity Disorder.

We conducted data synthesis in a semi-quantitative manner. We graded each health outcome in the included studies using the GRADE system and assessed their statistical significance. If for the same or similar outcomes, there was consistent statistical significance across all studies, we considered the result to be robust. This would indicate that green (or blue) is a protective factor for health. Conversely, we might not be able to confirm the consistency of the research, or for some outcomes, the studies show inconsistency, making it difficult to determine the association (Figure 1; Table 1; Supplementary Table S4). All the meta-analyses referenced in the results section are included in the umbrella review.

**Table 1:** Main findings of the included systematic reviews with meta-analyses (*N* = 47).

Authors	Published year	Participants	Interventions/exposure indicators	Outcome	Types of included studies	Number of included studies	Effect size and value	95% CI	Heterogeneity	Statistically significant
E. Fuertes, et al.	2016	Children (aged 6–8 years)	NDVI in 500 m	Allergic Rhinitis Incidence	Observational	6	OR = 1.00	0.69, 1.45	I^2^ = 81.2%	N
		Children (aged 6–8 years)	NDVI in 500 m	Aeroallergen Sensitization Incidence	Observational	6	OR = 0.96	0.75, 1.22	I^2^ = 73.8%	N
		Children (aged 10–12 years)	NDVI in 500 m	Allergic Rhinitis Incidence	Observational	5	OR = 0.96	0.71, 1.30	I^2^ = 76.9%	N
		Children (aged 10–12 years)	NDVI in 500 m	Aeroallergen Sensitization Incidence	Observational	5	OR = 0.85	0.61, 1.18	I^2^ = 76.5%	N
K.A. Lambert, et al.	2017	Children and adolescents (aged≤18)	NDVI	Asthma Prevalence	Observational	4	OR = 1.01	0.93, 1.09	I^2^ = 68.1%	N
			NDVI	Allergic Rhinitis Prevalence	Observational	6	OR = 0.99	0.87, 1.12	I^2^ = 72.9%	N
Caoimhe Twohig-Bennett, et al.	2018	General population	Green space exposure	Good Self-reported Health	Interventional and Observational	10	OR = 1.12	1.05, 1.19	I^2^ = 1	Y
			Green space exposure	Preterm Birth	Interventional and Observational	6	OR = 0.87	0.80, 0.94	I^2^ = 68%	Y
			Green space exposure	Type 2 Diabetes	Interventional and Observational	6	OR = 0.72	0.61, 0.85	I^2^ = 73%	Y
			Green space exposure	All-Cause Mortality	Interventional and Observational	4	OR = 0.69	0.55, 0.87	I^2^ = 96%	Y
			Green space exposure	Hypertension	Interventional and Observational	4	OR = 0.99	0.81, 1.20	I^2^ = 62%	N
			Green space exposure	Small for Gestational Age	Interventional and Observational	4	OR = 0.81	0.76, 0.86	I^2^ = 65%	Y
			Green space exposure	Cardiovascular Mortality	Interventional and Observational	2	OR = 0.84	0.76, 0.93	I^2^ = 54%	Y
			Green space exposure	Stroke	Interventional and Observational	3	OR = 0.82	0.61, 1.11	I^2^ = 59%	N
			Green space exposure	Dyslipidaemia	Interventional and Observational	2	OR = 0.94	0.75, 1.17	I^2^ = 57%	N
			Green space exposure	Asthma	Interventional and Observational	2	OR = 0.93	0.57, 1.52	I^2^ = 68%	N
			Green space exposure	Coronary Heart Disease	Interventional and Observational	2	OR = 0.92	0.78, 1.07	I^2^ = 48%	N
N. R. den Braver, et al.	2018(2021revised)	Adults (aged≥18)	More greenness	Type 2 Diabetes Mellitus Risk	Observational	4	OR = 0.91	0.88, 0.95	I^2^ = 0	Y
David Rojas-Rueda, et al.	2019	General population	NDVI in 500 m	All-cause Mortality	Observational	13	HR = 0.96	0.94, 0.97	I^2^ = 95%	Y
Hannah Roberts, et al.	2019	General population	Exposure to the natural environment	Depression Risk	Interventional and Observational	33	Mean effect size = −0.29	−2.30, 0.84	Qresid = 277.97, *p* < 0.01	N
Michele Antonelli, et al.	2019	General population	Activities conducted physically in the forest or natural settings	Cortisol as a Stress Biomarker	Interventional	10	Mean Difference = −0.05	−0.06, −0.04	I^2^ = 88%	Y
Ya-Na Luo, et al.	2020	General population	NDVI	Obesity Risk	Observational	6	RR = 0.88	0.84, 0.91	I^2^ = 38.95%	Y
		General population	Proximity to the nearest greenspace	Obesity Risk	Observational	4	RR = 0.99	0.99,1.00	I^2^ = 0	Y
		General population	Proportion of greenspace	Obesity Risk	Observational	6	RR = 0.96	0.85,1.08	I^2^ = 0.9732	N
		General population	Number of parks in an area	Obesity Risk	Observational	5	RR = 0.98	0.97,1.00	I^2^ = 0	Y
Eija Parmes, et al.	2020	Children (aged 3–14 years)	Living close to a coniferous forest	Wheezing Risk	Observational	9	OR = 1.06	0.98, 1.15	High	N
			Living close to a coniferous forest	Current Wheezing Risk	Observational	9	OR = 1.76	1.05, 2.97	Concern	Y
			Living close to a coniferous forest	Lifetime Wheezing Risk	Observational	9	OR = 3.95	2.08, 7.49	Concern	Y
			Living close to a coniferous forest	Current Asthma Risk	Observational	9	OR = 4.45	1.81, 10.9	Concern	Y
			Living close to a coniferous forest	Lifetime Asthma Risk	Observational	9	OR = 2.54	1.50, 5.82	Concern	Y
			Living close to a coniferous forest	Allergic Rhinitis Risk	Observational	9	OR = 3.39	1.83, 6.30	Concern	Y
Kyung Ju Lee, et al.	2020	Mothers and newborns	NDVI in 100, 250 and 500 m	Birth Weight	Observational	17	Pooled Regression Slope = 0.00134	0.000, 0.0020	I^2^ = 81%	Y
			NDVI in 100, 250 and 500 m	LBW + SGA Incidence	Observational	16	OR = 0.94	0.92, 0.97	I^2^ = 78%	Y
			NDVI in 100, 250 and 500 m	Preterm Birth Incidence	Observational	11	OR = 0.98	0.97, 0.99	I^2^ = 0	Y
Yongle Zhan, et al.	2020	Mothers and newborns	NDVI in 100 m	Birth Weight	Observational	36	β = 20.22	13.50, 26.93	I^2^ = 93.2%	Y
			NDVI in 100 m	Low Birth Weight (LBW) Risk	Observational	36	OR = 0.86	0.75, 0.99	I^2^ = 83.8%	Y
			NDVI in 100 m	Small for Gestational Age (SGA) Risk	Observational	36	OR = 0.93	0.88, 1.00	I^2^ = 24.2%	Y
Selin Akaraci, et al.	2020	Mothers and newborns	NDVI; Green space (land use data)	Birth Weight	Observational	22	β = 0.001	0.0002,0.002	I^2^ = 86%	Y
			NDVI; Green space (land use data)	Small for Gestational Age Risk	Observational	13	OR = 0.95	0.92, 0.97	I^2^ = 0.279	Y
			NDVI; Green space (land use data)	Low Birth Weight Risk	Observational	10	OR = 0.96	0.91, 1.01	I^2^ = 83.3%	N
			NDVI; Green space (land use data)	Preterm Birth Risk	Observational	11	OR = 0.99	0.97, 1.02	I^2^ = 53.58%	N
Giuseppina Spano, et al.	2020	Adults and elders	Community gardening or horticultural intervention	Psychosocial Well-Being	Interventional and Observational	11	SMD = 0.35	0.13, 0.56	High	Y
C. Bertrand, et al.	2021	General population for adults	NDVI	All-Cause Mortality	Observational	13	RR = 0.96	0.94, 0.97	no excess significance bias	Y
			NDVI	Cardiovascular Disease Mortality	Observational	10	RR = 0.98	0.96, 0.99	no excess significance bias	Y
			NDVI	Respiratory Disease Mortality	Observational	5	RR = 0.97	0.92; 1.02	no excess significance bias	N
KP Kua, et al.	2021	General population	Different quartiles of green spaces	All-cause Mortality	Observational	11	HR = 0.97	0.93, 1.02	I^2^ = 87.8%	N
Yin Yuan, et al.	2021	Older adult (aged≥60)	NDVI	All-cause Mortality	Observational	8	HR = 0.99	0.97, 1.00	I^2^ = 87.6%	Y
			NDVI	Cardiovascular Disease Mortality	Observational	4	HR = 0.99	0.99, 1.09	I^2^ = 76.4%	N
			NDVI	Ischemic Heart Disease Mortality	Observational	3	HR = 0.96	0.88, 1.05	I^2^ = 54.6%	N
			NDVI	Respiratory Disease Mortality	Observational	5	HR = 0.99	0.89, 1.00	I^2^ = 64.6%	Y
			NDVI	Stroke Mortality	Observational	4	HR = 0.77	0.59, 1.00	I^2^ = 78.8%	Y
Yong-Li Zhao, et al.	2021	Adults (aged≥18)	More greenness	Dementia Incidence	Observational	8	OR = 0.97	0.95, 0.995	I^2^ = 56.5%	Y
Cheng-Yang Hu, et al.	2021	Mothers and newborns	NDVI	Birth Weight	Observational	15	β = 13.42	6.57, 20.27	I^2^ = 90.7%	Y
			NDVI	Small for Gestational Age Risk	Observational	7	OR = 1	0.91, 1.09	I^2^ = 57.9%	N
			NDVI	Preterm Birth Risk	Observational	7	OR = 0.99	0.97, 1.00	I^2^ = 16.2%	Y
			NDVI	Low Birth Weight Risk	Observational	8	OR = 0.9	0.83, 0.99	I^2^ = 69.8%	Y
Zaeema Ahmer, et al.	2021	Mothers and newborns	NDVI in 250 m	Birth Weight	Observational	9	β = 8.95	1.63, 16.27	I^2^ = 88.95%	Y
			NDVI in 250 m	Low Birth Weight Risk	Observational	6	OR = 0.97	0.96, 0.98	I^2^ = 90.3%	Y
Peter A. Coventry, et al.	2021	General population	Nature-based outdoor activities	Improving Depressive Mood Incidence	Interventional	50	SMD = -0.64	−1.05, −0.23	I^2^ = 85.7%	Y
			Nature-based outdoor activities	Reducing Anxiety Incidence	Interventional	50	SMD = -0.94	−1.87, −0.01	I^2^ = 93.7%	Y
			Nature-based outdoor activities	Improving Positive Affect Incidence	Interventional	50	SMD = 0.95	0.59, 1.31	I^2^ = 45.8%	Y
			Nature-based outdoor activities	Reducing Negative Affect Incidence	Interventional	50	SMD = -0.52	−0.77, −0.26	I^2^ = 9.8%	Y
Wenfei Yao, et al.	2021	Adults (aged≥18)	Exposure to the natural environment	Positive Affect	Interventional and Observational	20	SMD = 0.61	0.41, 0.81	I^2^ = 78.4%	Y
J Mark Noordzij, et al.	2021	Adults (aged 50–71 years)	NDVI in 800 m or the distance to the nearest green space	Good Self-rated Health	Observational	4	OR = 1.01	0.99, 1.02	NA	N
			NDVI in 800 m or the distance to the nearest green space	Depression Prevalence	Observational	4	OR = 0.98	0.96, 1.00	NA	N
Niamh Smith, et al.	2021	General Population	Exposure to all types of urban blue space	All-cause Mortality	Observational	3	HR = 0.99	0.97,1.00	I^2^ > 75%	Y
			Exposure to all types of urban blue space	Obesity Risk	Observational	3	β = −0.34	−0.19, -0.09	I^2^ > 75%	Y
			Exposure to all types of urban blue space	Good Self-rated Health	Observational	4	Cohen’s d = −0.09	−0.10, -0.08	I^2^ > 75%	Y
			Exposure to all types of urban blue space	Mental Health and Wellbeing	Observational	4	Cohen’s d = −0.25	−0.44, -0.07	I^2^ > 75%	Y
Xiao-Xuan Liu, et al.	2022	General population	NDVI in 300 m	Cardiovascular Disease Mortality	Observational	10	OR = 0.97	0.96, 0.99	Q = 225.04	Y
			NDVI in 300 m	Ischemic Heart Disease Mortality	Observational	8	OR = 0.98	0.96, 1.00	Q = 73.40	Y
			NDVI in 300 m	Cerebrovascular Disease Mortality	Observational	6	OR = 0.98	0.97, 1.00	Q = 13.40	Y
			NDVI in 300 m	Stroke Incidence	Observational	3	OR = 0.98	0.96, 0.99	Q = 4.00	Y
Yu Zhao, et al.	2022	General population	NDVI; Proportion of Greenness; Distance to greenness	Systolic Blood Pressure	Observational	4	β = −0.77	−1.23, −0.32	I^2^ = 94%	Y
			NDVI; Proportion of Greenness; Distance to greenness	Diastolic Blood Pressure	Observational	4	β = −0.32	−0.57, −0.07	I^2^ = 88%	Y
Mohammad Javad Zare Sakhvidi, et al.	2022	General population	Preferred NDVI in 300 m	All-site Cancer Mortality	Observational	18	Not statistically significant	Pooled estimates	NA	N
			Preferred NDVI in 300 m	All-site Cancer Incidence	Observational	18	Not statistically significant	Pooled estimates	NA	N
			Preferred NDVI in 300 m	Lung Cancer Mortality	Observational	9	OR = 0.99	0.84, 1.20	I^2^ = 0	N
Federico Zagnoli, et al.	2022	Adults (aged≥18)	NDVI	Dementia Incidence and Mortality	Observational	7	RR = 0.98	0.90, 1.06	I^2^ = 97.54%	N
			Land Use/Cover (LU/LC)	Dementia Incidence and Mortality	Observational	6	RR = 0.99	0.93, 1.05	I^2^ = 81.48%	N
Birong Wu, et al.	2022	General population for adults	NDVI in 100 m	Current Asthma Incidence	Observational	3	OR = 0.98	0.90, 1.07	I^2^ = 30.1%	N
			NDVI in 100-300 m	Current Asthma Incidence	Observational	6	OR = 0.99	0.91, 1.07	I^2^ = 0	N
			NDVI in 300-500 m	Current Asthma Incidence	Observational	6	OR = 1	0.91, 1.09	I^2^ = 0	N
			NDVI in 500-1000 m	Current Asthma Incidence	Observational	6	OR = 0.98	0.90, 1.08	I^2^ = 0	N
			NDVI in 100 m	Ever Asthma Incidence	Observational	4	OR = 1.04	0.92, 1.16	I^2^ = 70.2%	N
			NDVI in 100-300 m	Ever Asthma Incidence	Observational	4	OR = 1	0.99, 1.02	I^2^ = 0	N
			NDVI in 300-500 m	Ever Asthma Incidence	Observational	3	OR = 1.04	0.90, 1.22	I^2^ = 0	N
			NDVI in 100 m	Allergic Rhinitis Incidence	Observational	3	OR = 0.98	0.95, 1.02	I^2^ = 0	N
			NDVI in 500 m	Allergic Rhinitis Incidence	Observational	5	OR = 0.99	0.94, 1.04	I^2^ = 0	N
			NDVI in 1000 m	Allergic Rhinitis Incidence	Observational	3	OR = 1	0.95, 1.05	I^2^ = 0	N
Song Song, et al.	2022	General Population	Green space exposure	Depression Risk	Interventional	9	ES = -0.50	−0.82, −0.18	I^2^ = 83.4%	Y
			Green space exposure	Negative Affect	Interventional	6	ES = -0.34	−0.61, −0.07	I^2^ = 53.5%	Y
			Green space exposure	Positive Affect	Interventional	6	ES = 0.57	0.24, 0.86	I^2^ = 58.7%	Y
Michail Georgiou, et al.	2022	Adults (aged≥18)	Distance to blue space	Physical Activity	Interventional and Observational	12	Cohen’s d = 0.122	0.065, 0.179	I^2^ = 99.49%	Y
			Amount of blue space around a certain geographical area	Physical Activity	Interventional and Observational	10	Cohen’s d = 0.144	0.024, 0.264	I^2^ = 99.34%	Y
			Distance to blue space	Restoration	Interventional and Observational	6	Cohen’s d = 0.123	−0.337, 0.284	I^2^ = 96.6%	N
			Amount of blue space around a certain geographical area	Restoration	Interventional and Observational	8	Cohen’s d = 0.339	0.072, 0.606	I^2^ = 91.97%	Y
			Contact with blue space	Restoration	Interventional and Observational	11	Cohen’s d = 0.191	0.084, 0.298	I^2^ = 79.5%	Y
			Distance to blue space	Social Interaction	Interventional and Observational	4	Cohen’s d = −0.214	−0.55, 0.122	I^2^ = 90.81%	N
			Amount of blue space around a certain geographical area	Social Interaction	Interventional and Observational	4	Cohen’s d = 0.405	−0.214, 1.024	I^2^ = 56.41%	N
Masashi Soga, et al.	2022	Adults (aged≥18)	Outdoor gardening	Health Variables	Interventional	18	Hedges’ d = 0.31	0.21, 0.40	*p* = 0.04	Y
			Outdoor gardening	Wellbeing Variables	Interventional	58	Hedges’ d = 0.47	0.39, 0.54	P<0.001	Y
			Outdoor gardening	Beneficial for Health	Interventional	76	Hedges’ d = 0.42	0.36, 0.48	I^2^ = 40.47%	Y
Alessandro Bianconi, et al.	2023	General population	NDVI and LAI	Cardiovascular Disease Mortality	Observational	6	HR = 0.94	0.91, 0.97	I^2^ = 97%	Y
			NDVI and LAI	Ischemic Heart Disease Mortality	Observational	5	HR = 0.96	0.93, 0.99	I^2^ = 90%	Y
			NDVI and LAI	Cerebrovascular Disease Mortality	Observational	5	HR = 0.96	0.94, 0.97	I^2^ = 23%	Y
Jiang L, et al.	2023	Adults (aged≥18)	NDVI	All-site Cancer Incidence	Observational	14	HR = 0.980	0.954, 1.006	I^2^ = 66.5%	N
			NDVI	All-site Cancer Mortality	Observational	13	HR = 0.962	0.946, 0.979	I^2^ = 80.0%	Y
			NDVI	Lung Cancer Incidence	Observational	3	HR = 0.903	0.801, 1.018	I^2^ = 66.8%	N
			NDVI	Lung Cancer Mortality	Observational	4	HR = 0.965	0.947, 0.983	I^2^ = 84.4%	Y
Fangzheng Li, et al.	2023	General population	NDVI	Parkinson Disease Incidence	Observational	3	RR = 0.89	0.78, 1.02	I^2^ = 93.5%	N
			NDVI	Stroke Incidence	Observational	4	RR = 0.98	0.97, 0.99	I^2^ = 45.8%	Y
			NDVI	Cerebrovascular Disease Mortality	Observational	4	RR = 0.98	0.97, 1.00	I^2^ = 79.9%	Y
			NDVI	Neurodegenerative Disease Mortality	Observational	3	RR = 0.98	0.98, 0.99	I^2^ = 0	Y
			NDVI	Stroke Mortality	Observational	3	RR = 0.96	0.93, 1.00	I^2^ = 58.7%	Y
Xue Wang, et al.	2023	General Population	NDVI	Current Asthma Risk	Observational	14	OR = 0.94	0.88, 1.00	I^2^ = 35%	Y
		Children	NDVI	Asthma Risk	Observational	8	OR = 0.94	0.94, 0.98	I^2^ = 25%	Y
		Children	NDVI	Allergic Rhinitis Risk	Observational	7	OR = 0.83	0.72, 0.96	I^2^ = 0	Y
Mingcheng Tang, et al.	2023	General Population	NDVI	Asthma Incidence	Observational	9	RR = 0.92	0.85, 0.98	I^2^ = 78%	Y
			NDVI	Allergic Rhinitis Incidence	Observational	6	RR = 1.02	0.97, 1.08	I^2^ = 65%	N
			NDVI	COPD Incidence	Observational	2	RR = 0.92	0.83, 1.03	I^2^ = 91%	N
			NDVI	COPD Mortality	Observational	3	RR = 0.95	0.92, 0.99	I^2^ = 7%	Y
			NDVI	Lung Cancer Incidence	Observational	5	RR = 0.62	0.40, 0.95	I^2^ = 97%	Y
			NDVI	Lung Cancer Mortality	Observational	6	RR = 0.98	0.96, 1.01	I^2^ = 88%	N
Nv-Wei Cao, et al.	2023	Children and adolescents (aged≤18)	NDVI	Allergic Rhinitis Risk	Observational	14	OR = 1.00	0.99, 1.00	I^2^ = 50.4%	N
Ziquan Liu, et al.	2023	General Population	Per 10% increase in percentage of green space	Depression Risk	Observational	13	OR = 0.963	0.948, 0.979	I^2^ = 65.5%	Y
			NDVI	Depression Risk	Observational	13	OR = 0.931	0.887, 0.977	I^2^ = 94.4%	Y
			Per 10% increase in percentage of green space	Anxiety Risk	Observational	3	OR = 0.938	0.858, 1.025	I^2^ = 81.5%	N
Rebecca Briggs, et al.	2023	Adults (aged≥18)	Outdoor gardening intervention	Depression Risk	Interventional	8	SMD = -0.43	−0.79, −0.06	I^2^ = 63%	Y
			Outdoor gardening intervention	Anxiety Risk	Interventional	5	SMD = -0.42	−1.00, 0.16	I^2^ = 81%	N
			Outdoor gardening intervention	Stress	Interventional	3	SMD = -0.17	−0.68, 0.35	I^2^ = 38%	N
			Outdoor gardening intervention	Health-related Quality of Life	Interventional	8	SMD = -0.06	−0.45, 0.34	I^2^ = 65%	N
			Outdoor gardening intervention	Psychosocial Well-Being	Interventional	4	SMD = 0.37	0.01, 0.73	I^2^ = 43%	Y
Chiew Jiat Rosalind Siah, et al.	2023	General Population	Activities conducted physically in the forest or natural settings	Depression Risk	Interventional	10	SMD = -0.67	−0.99, −0.35	I^2^ = 69%	Y
			Activities conducted physically in the forest or natural settings	Anxiety Risk	Interventional	6	SMD = -0.84	−1.42, −0.25	I^2^ = 84%	Y
			Activities conducted physically in the forest or natural settings	Systolic Blood Pressure	Interventional	13	MD = -1.66	−4.30, −0.97	I^2^ = 52%	Y
			Activities conducted physically in the forest or natural settings	Diastolic Blood Pressure	Interventional	13	MD = -3.09	−7.52, 1.34	I^2^ = 92%	N
			Activities conducted physically in the forest or natural settings	Heart Rate	Interventional	5	MD = -0.42	−3.32, 2.49	I^2^ = 57%	N
Yasaman Sharif, et al.	2024	General population	Self-reported frequency of visits	Diabetes Mellitus Risk	Observational	3	OR = 0.79	0.67, 0.90	I^2^ = 4.49%	Y
			Self-reported frequency of visits	Obesity Risk	Observational	3	OR = 0.83	0.77, 0.90	I^2^ = 66.92%	Y
			Self-reported frequency of visits	Hypertension Risk	Observational	16	OR = 0.81	0.61, 0.99	I^2^ = 83.1%	Y
Yongjun Bu, et al.	2024	General population for adults	NDVI in 1000 m	Systolic Blood Pressure	Observational	3	β = −1.41	‘-2.57--0.25	I^2^ = 96.8%	Y
			NDVI in 500 m	Systolic Blood Pressure	Observational	4	β = −1.32	−2.18--0.45	I^2^ = 94.9%	Y
			NDVI in 1000 m	Hypertension Risk	Observational	3	OR = 0.95	0.90, 0.99	I^2^ = 81.6%	Y
			NDVI in 500 m	Hypertension Risk	Observational	4	OR = 0.95	0.90, 0.99	I^2^ = 85.1%	Y
Muhammad Mainuddin Patwary, et al.	2024	General population	0.1NDVI in 500 m	Metabolic Syndrome Risk	Observational	4	OR = 0.90	0.87, 0.93	I^2^ = 23.8%	Y
Giulia Squillacioti, et al.	2024	Children and adolescents (aged≤18)	NDVI in 500 m	Asthma Incidence	Observational	3	OR = 0.97	0.53, 1.78	I^2^ = 54%	N
			High vs. low tertile of NDVI in 300 m	Asthma Incidence	Observational	3	OR = 0.65	0.22, 1.91	I^2^ = 74%	N
Yimin Zhang, et al.	2024	General Population	NDVI, area of green space, green spaces accessibility, parks, and other exposure index.	Psychiatric Disorders Risk	Observational	59	OR = 0.91	0.89, 0.92	I^2^ = 83.7%	Y
			NDVI, area of green space, green spaces accessibility, parks, and other exposure index.	Depression Risk	Observational	37	OR = 0.89	0.86, 0.93	I^2^ = 87.4%	Y
			NDVI	Anxiety Risk	Observational	14	OR = 0.94	0.92, 0.96	I^2^ = 57.2%	Y
			NDVI	Dementia Risk	Observational	8	OR = 0.95	0.93, 0.96	I^2^ = 52%	Y
			NDVI	Schizophrenia Risk	Observational	7	OR = 0.74	0.67, 0.82	I^2^ = 60.6%	Y
			NDVI	ADHD Risk	Observational	5	OR = 0.89	0.86, 0.92	I^2^ = 37.3%	Y
Tim Cadman, et al.	2024	Mothers without a previous history of depression	NDVI	Postpartum Depression Risk	Observational	12	OR = 0.99	0.93, 1.05	I^2^ = 0	N
			Major Green Space	Postpartum Depression Risk	Observational	12	OR = 0.98	0.89, 1.07	I^2^ = 2.22%	N
			Major Blue Space	Postpartum Depression Risk	Observational	12	OR = 1.12	1.00, 1.26	I^2^ = 0	Y
I. Pantiru, et al.	2024	Adults and specific clinical populations	Gardening or Horticultural therapy	Psychosocial Well-being	Interventional and Observational	6	Effect size = 0.55	0.23, 0.87	I^2^ = 88.5%	Y

"Interventions/exposure indicators" column, in order to highlight the results of the blue space, BLUE was used to emphasize the exposure outcomes of the blue space. In the column of "Types of included studies", we use DARK GREEN to represent the results of single Interventional studies, LIGHTER GREEN to indicate the results of both containing interventional and observational studies, while LIGHT GREEN to represent the results of single observational studies. In the "Heterogeneity" column, to facilitate the distinction of heterogeneity, we used different shades of green for differentiation. DARK GREEN indicates high heterogeneity, LIGHTER GREEN indicates moderate heterogeneity, while LIGHT GREEN indicates low heterogeneity. Unmarked colors indicate that heterogeneity is not mentioned in the literature, and its validity deserves attention.

Key details are extracted, including author, year of publication, type of study design (observational or interventional), key findings, and defining features of the major studies included. These features include age range, sample size, methods for assessing green/blue spaces, health outcomes, effect size, 95% confidence intervals, and statistical significance. Heterogeneity was also included in the statistics, and was mainly assessed using an I^2^ value: where 0–30% was defined as low heterogeneity, 30–70% as moderate heterogeneity, and more than 70% as high heterogeneity. If high heterogeneity is observed, the confidence with the evidence is reduced.

## 3 Results

### 3.1 Systematic review retrieval

**Figure 2:**
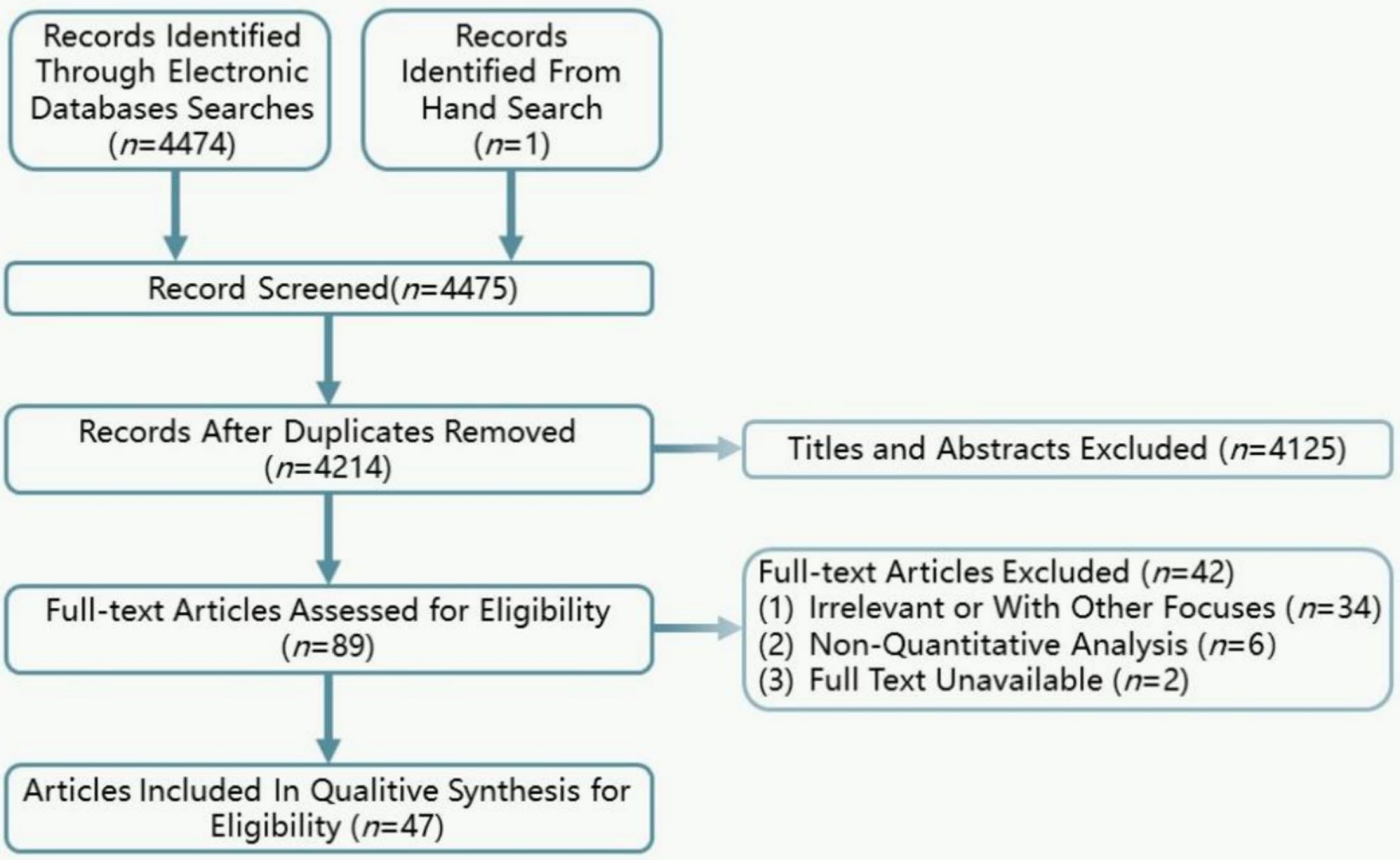
Flow chart of study selection process.

The initial search identified 4,475 records. After removing duplicates, 4,214 titles and abstracts of systematic reviews were assessed, and 4,125 articles were excluded during the title and abstract screening, 89 articles were subjected to full-text review. A total of 34 articles were further eliminated as they were irrelevant to the topic or focused on other priorities. Two articles (19, 20) were excluded due to unavailability of full text or being conference abstracts, and 6 articles (21–26) were excluded for not being quantitative analyses. Finally, 47 meta-analyses were included in the umbrella review (Figure 2).

### 3.2 Characteristics of systematic reviews included in the umbrella review

The umbrella review included 47 systematic reviews with meta-analyses. Most of these articles were published between 2021 and 2024, with 34 articles (approximately 72%) published after 2021. The number of primary studies included in the meta-analyses for each health outcome ranged from 2 to 76. Most of the primary studies incorporated were observational, with experimental or interventional studies in the minority. Our research spanned a wide demographic range, encompassing individuals from infancy to old age, and was predominantly centered on regions known for their capacity to execute extensive cohort studies, including North America, Europe, and China.

### 3.3 Green/blue spaces exposure measures

Of the 47 studies, 45 were related to green spaces. The remaining two assessed blue spaces. Various metrics have been used to assess green space exposure. Objective parameters include the Normalized Difference Vegetation Index (NDVI) (29/45), Leaf Area Index (LAI) (1/46), area of green patches (6/45), distance to the nearest green space (2/45), and the number of nearby parks (1/45). One quantitative review analyzing the health impacts of residential building characteristics included descriptions of land use types (1/45). Subjective parameters included self-reported exposure and visits to natural environments (8/45). We separately assessed activities conducted in natural environments, such as forest bathing (2/45) and gardening (4/45). Blue space exposure assessments included distance, the presence of blue spaces within specific buffer zones, blue space coverage, and self-reported frequency of use.

### 3.4 Health outcomes

We categorized the health outcomes related to green space exposure into several sections: outcomes associated with green space exposure include mortality, neurological disorders and cognitive function, cardiovascular and metabolic diseases (including cardiovascular diseases, diabetes, metabolic syndrome, overweight and obesity, metabolic indicators), cancer, allergic diseases (mainly affecting children and adolescents), pregnancy outcomes, and mental health. Additionally, we conducted a separate review of blue space outcomes. These health outcomes were measured using various methods, including physician diagnosis, questionnaire surveys, records from hospitals or other health-related departments, self-reported health status, and laboratory tests.

### 3.5 Methodological quality

Many of the included systematic reviews did not meet all seven key domains of the AMSTAR2 checklist above (Table 2). 25 out of 47 reviews (53%) developed a protocol for this review. 46 out of 47 reviews (98%) conducted a comprehensive literature search or pre-specified specific cohorts. Of the 47 reviews, 11 (23%) offered a rationale for excluding studies, while 46 (98%) employed suitable meta-analysis techniques. A strong majority, 42 (89%), considered the potential bias in the primary studies during their discussions. Nevertheless, only 32 (68%) of the reviews evaluated the risk of publication bias due to small sample sizes in the primary research. We used GRADE grading to assess each study’s evidence level for each health outcome (N = 154). Most of the evidence from studies ranged from “very low” to “low” quality (Table 3).

**Table 2:** Methodological quality based on AMSTAR2 of included reviews with meta-analyses (*N* = 47).

Authors	Published year	AMSTAR#1	AMSTAR#2	AMSTAR#3	AMSTAR#4	AMSTAR#5	AMSTAR#6	AMSTAR#7	AMSTAR#8	AMSTAR#9	AMSTAR#10	AMSTAR#11	AMSTAR#12	AMSTAR#13	AMSTAR#14	AMSTAR#15	AMSTAR#16
E. Fuertes, et al.	2016	Y	N	Y	Y	N	N	N	Y	Y	Y	Y	Y	Y	Y	N	Y
K.A. Lambert, et al.	2017	Y	N	Y	Y	Y	Y	N	Y	Y	N	Y	Y	Y	Y	N	Y
Caoimhe Twohig-Bennett, et al.	2018	Y	Y	Y	Y	Y	Y	P	Y	Y	N	Y	Y	Y	Y	Y	Y
N. R. den Braver, et al.	2018 (2021 revised)	Y	Y	Y	Y	Y	Y	P	Y	Y	N	Y	Y	Y	Y	Y	Y
David Rojas-Rueda, et al.	2019	Y	Y	Y	Y	Y	Y	Y	Y	Y	N	Y	Y	Y	Y	Y	Y
Hannah Roberts, et al.	2019	Y	Y	Y	Y	N	N	N	Y	Y	N	Y	Y	Y	Y	Y	Y
Michele Antonelli, et al.	2019	Y	N	Y	Y	Y	Y	N	Y	Y	N	Y	Y	Y	Y	Y	N
Ya-Na Luo, et al.	2020	Y	N	Y	Y	Y	Y	Y	Y	Y	N	Y	Y	Y	Y	P	Y
Eija Parmes, et al.	2020	Y	N	Y	Y	N	N	N	Y	Y	Y	Y	Y	Y	Y	N	Y
Kyung Ju Lee, et al.	2020	Y	N	Y	Y	Y	Y	N	Y	Y	N	Y	Y	Y	Y	Y	Y
Yongle Zhan, et al.	2020	Y	N	Y	Y	Y	Y	N	Y	P	N	Y	P	P	Y	Y	Y
Selin Akaraci, et al.	2020	Y	N	Y	Y	Y	Y	P	Y	Y	N	Y	Y	Y	Y	Y	Y
Giuseppina Spano, et al.	2020	Y	N	Y	Y	Y	Y	N	Y	Y	N	Y	Y	Y	Y	Y	Y
C. Bertrand, et al.	2021	Y	P	Y	Y	N	N	N	Y	Y	N	Y	Y	Y	N	N	Y
KP Kua, et al.	2021	Y	N	Y	Y	Y	Y	N	Y	Y	N	Y	P	Y	Y	N	Y
Yin Yuan, et al.	2021	Y	Y	Y	Y	Y	Y	N	Y	Y	N	Y	P	Y	Y	Y	Y
Yong-Li Zhao, et al.	2021	Y	Y	Y	Y	Y	Y	Y	Y	Y	N	Y	Y	Y	Y	Y	Y
Cheng-Yang Hu, et al.	2021	Y	N	Y	Y	Y	Y	N	Y	Y	Y	Y	Y	Y	Y	Y	Y
Zaeema Ahmer, et al.	2021	Y	Y	Y	Y	Y	Y	N	Y	Y	N	Y	Y	Y	Y	Y	Y
Peter A. Coventry, et al.	2021	Y	Y	Y	Y	Y	Y	N	Y	Y	N	Y	Y	Y	Y	Y	Y
Wenfei Yao, et al.	2021	Y	Y	Y	Y	Y	Y	Y	Y	Y	N	Y	Y	Y	Y	Y	Y
J Mark Noordzij, et al.	2021	Y	Y	Y	Y	N	N	N	Y	Y	Y	Y	Y	Y	Y	N	Y
Niamh Smith, et al.	2021	Y	Y	Y	Y	Y	Y	N	Y	P	N	Y	P	Y	Y	N	Y
Xiao-Xuan Liu, et al.	2022	Y	N	Y	Y	Y	Y	Y	Y	Y	N	Y	Y	Y	Y	Y	Y
Yu Zhao, et al.	2022	Y	Y	Y	Y	Y	Y	Y	Y	Y	N	Y	Y	Y	Y	Y	Y
Mohammad Javad Zare Sakhvidi, et al.	2022	Y	Y	Y	Y	Y	Y	Y	Y	Y	Y	Y	Y	Y	Y	Y	Y
Federico Zagnoli, et al.	2022	Y	N	Y	Y	Y	Y	Y	Y	Y	N	Y	Y	Y	Y	N	Y
Birong Wu, et al.	2022	Y	Y	Y	Y	Y	Y	N	Y	Y	N	Y	Y	Y	Y	Y	Y
Song Song, et al.	2022	Y	Y	Y	Y	Y	Y	N	Y	Y	N	Y	Y	Y	Y	Y	Y
Michail Georgiou, et al.	2022	Y	Y	Y	Y	Y	Y	N	Y	Y	N	Y	Y	Y	Y	N	Y
Masashi Soga, et al.	2022	Y	N	Y	Y	Y	Y	N	Y	Y	N	Y	Y	Y	Y	Y	Y
Alessandro Bianconi, et al.	2023	Y	Y	Y	Y	Y	Y	Y	Y	Y	N	Y	Y	Y	Y	Y	Y
Jiang L, et al.	2023	Y	Y	Y	Y	Y	Y	Y	Y	Y	Y	N	P	Y	Y	Y	Y
Fangzheng Li, et al.	2023	Y	Y	Y	Y	Y	Y	N	Y	Y	N	Y	Y	Y	Y	Y	Y
Xue Wang, et al.	2023	Y	N	Y	Y	Y	Y	N	Y	Y	N	Y	Y	Y	Y	Y	Y
Mingcheng Tang, et al.	2023	Y	Y	Y	Y	Y	Y	N	Y	Y	N	Y	Y	Y	Y	Y	Y
Nv-Wei Cao, et al.	2023	Y	N	Y	Y	Y	Y	Y	Y	Y	Y	Y	Y	Y	Y	Y	Y
Ziquan Liu, et al.	2023	Y	N	Y	Y	Y	Y	N	Y	Y	N	Y	Y	Y	Y	Y	Y
Rebecca Briggs, et al.	2023	Y	Y	Y	Y	Y	Y	N	Y	Y	N	Y	Y	Y	Y	P	Y
Chiew Jiat Rosalind Siah, et al.	2023	Y	N	Y	Y	Y	Y	N	Y	Y	N	Y	Y	Y	Y	N	Y
Yasaman Sharif, et al.	2024	Y	Y	Y	Y	Y	Y	N	Y	Y	N	Y	Y	Y	Y	Y	Y
Yongjun Bu, et al.	2024	Y	N	Y	Y	Y	Y	N	Y	Y	N	Y	Y	Y	Y	Y	Y
Muhammad Mainuddin Patwary, et al.	2024	Y	Y	Y	Y	Y	Y	N	Y	Y	N	Y	Y	Y	Y	Y	Y
Giulia Squillacioti, et al.	2024	Y	Y	Y	Y	Y	Y	P	Y	Y	N	Y	Y	Y	Y	N	Y
Yimin Zhang, et al.	2024	Y	N	Y	Y	Y	Y	N	Y	Y	N	Y	Y	Y	Y	N	Y
Tim Cadman, et al.	2024	Y	Y	Y	P	N	N	N	Y	P	Y	Y	P	P	Y	N	Y
I. Panțiru, et al.	2024	Y	N	Y	Y	Y	Y	N	Y	Y	N	Y	Y	Y	Y	Y	Y

**Table 3:** GRADE assessment of the included systematic reviews with meta-analyses (*N* = 47).

Authors	Published year	Outcome	Types of included studies	Risk of bias	Inconsistency	Indirectness	Imprecision	Dose–response	Certainty
E. Fuertes, et al.	2016	Allergic Rhinitis Incidence	Observational	Concern	High	Low	High	N	Very Low
		Aeroallergen Sensitization Incidence	Observational	Concern	High	Low	High	N	Very Low
		Allergic Rhinitis Incidence	Observational	Concern	High	Low	High	N	Very Low
		Aeroallergen Sensitization Incidence	Observational	Concern	High	Low	High	N	Very Low
K.A. Lambert, et al.	2017	Asthma Prevalence	Observational	Concern	Moderate	Low	High	N	Very Low
		Allergic Rhinitis Prevalence	Observational	Concern	High	Low	High	N	Very Low
Caoimhe Twohig-Bennett, et al.	2018	Good Self-reported Health	Interventional and Observational	Low	High	Low	Low	N	Low
		Preterm Birth	Interventional and Observational	Low	Moderate	Low	Low	N	Moderate
		Type 2 Diabetes	Interventional and Observational	Low	High	Low	Low	N	Low
		All-Cause Mortality	Interventional and Observational	Low	High	Low	Low	N	Low
		Hypertension	Interventional and Observational	Low	Moderate	Low	High	N	Low
		Small for Gestational Age	Interventional and Observational	Low	Moderate	Low	Low	N	Moderate
		Cardiovascular Mortality	Interventional and Observational	Low	Moderate	Low	Low	N	Moderate
		Stroke	Interventional and Observational	Low	Moderate	Low	High	N	Low
		Dyslipidaemia	Interventional and Observational	Low	Moderate	Low	High	N	Low
		Asthma	Interventional and Observational	Low	Moderate	Low	High	N	Low
		Coronary Heart Disease	Interventional and Observational	Low	Moderate	Low	High	N	Low
N. R. den Braver, et al.	2018 (2021 revised)	Type 2 Diabetes Mellitus Risk	Observational	Concern	Low	Low	Low	N	Very Low
David Rojas-Rueda, et al.	2019	All-cause Mortality	Observational	Low	High	Low	Low	N	Very Low
Hannah Roberts, et al.	2019	Depression Risk	Interventional and Observational	Concern	High	Low	High	N	Very Low
Michele Antonelli, et al.	2019	Cortisol as a Stress Biomarker	Interventional	Concern	High	Low	Low	N	Low
Ya-Na Luo, et al.	2020	Obesity Risk	Observational	Concern	Moderate	Low	Low	N	Very Low
		Obesity Risk	Observational	Concern	Low	Low	Low	N	Very Low
		Obesity Risk	Observational	Concern	High	Low	High	N	Very Low
		Obesity Risk	Observational	Concern	Low	Low	Low	N	Very Low
Eija Parmes, et al.	2020	Wheezing Risk	Observational	Low	High	Low	High	N	Very Low
		Current Wheezing Risk	Observational	Low	Concern	Low	Low	N	Very Low
		Lifetime Wheezing Risk	Observational	Low	Concern	Low	Low	N	Very Low
		Current Asthma Risk	Observational	Low	Concern	Low	Low	N	Very Low
		Lifetime Asthma Risk	Observational	Low	Concern	Low	Low	N	Very Low
		Allergic Rhinitis Risk	Observational	Low	Concern	Low	Low	N	Very Low
Kyung Ju Lee, et al.	2020	Birth Weight	Observational	Low	High	Low	Low	N	Very Low
		LBW + SGA Incidence	Observational	Low	High	moderate	Low	N	Very Low
		Preterm Birth Incidence	Observational	Low	Low	Low	low	N	Low
Yongle Zhan, et al.	2020	Birth Weight	Observational	Moderate	High	Low	Low	N	Very Low
		Low Birth Weight (LBW) Risk	Observational	Low	High	Low	Low	Y	Very Low
		Small for Gestational Age (SGA) Risk	Observational	Low	Low	Low	Low	Y	Low
Selin Akaraci, et al.	2020	Birth Weight	Observational	High	High	Low	Low	N	Very Low
		Small for Gestational Age Risk	Observational	High	Low	Low	Low	N	Very Low
		Low Birth Weight Risk	Observational	High	High	Low	High	N	Very Low
		Preterm Birth Risk	Observational	High	Moderate	Low	High	N	Very Low
Giuseppina Spano, et al.	2020	Psychosocial Well-Being	Interventional and Observational	High	High	High	Low	N	Very Low
C. Bertrand, et al.	2021	All-Cause Mortality	Observational	Concern	Concern	Low	Low	N	Very Low
		Cardiovascular Disease Mortality	Observational	Concern	Concern	Low	Low	N	Very Low
		Respiratory Disease Mortality	Observational	Concern	Concern	Low	High	N	Very Low
KP Kua, et al.	2021	All-cause Mortality	Observational	Concern	High	Low	Low	N	Very Low
Yin Yuan, et al.	2021	All-cause Mortality	Observational	Low	High	Low	Low	N	Very Low
		Cardiovascular Disease Mortality	Observational	Low	High	Low	High	N	Very Low
		Ischemic Heart Disease Mortality	Observational	Low	Moderate	Low	High	N	Very Low
		Respiratory Disease Mortality	Observational	Low	Moderate	Low	Low	N	Low
		Stroke Mortality	Observational	Low	High	Low	Low	N	Very Low
Yong-Li Zhao, et al.	2021	Dementia Incidence	Observational	Low	Moderate	Low	Low	N	Low
Cheng-Yang Hu, et al.	2021	Birth Weight	Observational	Low	High	Low	Low	N	Very Low
		Small for Gestational Age Risk	Observational	Low	Moderate	Low	High	N	Very Low
		Preterm Birth Risk	Observational	Low	Low	Low	Low	N	Low
		Low Birth Weight Risk	Observational	Low	Moderate	Low	Low	N	Low
Zaeema Ahmer, et al.	2021	Birth Weight	Observational	Low	High	Low	Low	N	Very Low
		Low Birth Weight Risk	Observational	Low	High	Low	Low	N	Very Low
Peter A. Coventry, et al.	2021	Improving Depressive Mood Incidence	Interventional	Low	High	Low	Low	N	Moderate
		Reducing Anxiety Incidence	Interventional	Low	High	Low	Low	N	Moderate
		Improving Positive Affect Incidence	Interventional	Low	Moderate	Low	Low	N	High
		Reducing Negative Affect Incidence	Interventional	Low	Low	Low	Low	N	High
Wenfei Yao, et al.	2021	Positive Affect	Interventional and Observational	Low	High	High	High	N	Very Low
J Mark Noordzij, et al.	2021	Good Self-rated Health	Observational	High	High	Low	High	N	Very Low
		Depression Prevalence	Observational	High	High	Low	High	N	Very Low
Niamh Smith, et al.	2021	All-cause Mortality	Observational	Concern	High	Low	Low	N	Very Low
		Obesity Risk	Observational	Concern	High	Low	Low	N	Very Low
		Good Self-rated Health	Observational	Concern	High	Low	Low	N	Very Low
		Mental Health and Wellbeing	Observational	Concern	High	Low	Low	N	Very Low
Xiao-Xuan Liu, et al.	2022	Cardiovascular Disease Mortality	Observational	Low	High	Low	Low	N	Very Low
		Ischemic Heart Disease Mortality	Observational	Low	High	Low	Low	N	Very Low
		Cerebrovascular Disease Mortality	Observational	Low	Moderate	Low	Low	N	Low
		Stroke Incidence	Observational	Low	Low	Low	Low	N	Low
Yu Zhao, et al.	2022	Systolic Blood Pressure	Observational	Low	High	Low	Low	N	Very Low
		Diastolic Blood Pressure	Observational	Low	High	Low	Low	N	Very Low
Mohammad Javad Zare Sakhvidi, et al.	2022	All-site Cancer Mortality	Observational	Concern	High	High	High	N	Very Low
		All-site Cancer Incidence	Observational	Concern	High	High	High	N	Very Low
		Lung Cancer Mortality	Observational	Concern	Low	High	High	N	Very Low
Federico Zagnoli, et al.	2022	Dementia Incidence and Mortality	Observational	Concern	High	Low	High	N	Very Low
		Dementia Incidence and Mortality	Observational	Concern	High	Low	High	Y	Very Low
Birong Wu, et al.	2022	Current Asthma Incidence	Observational	Low	Moderate	Low	High	N	Very Low
		Current Asthma Incidence	Observational	Low	Low	Low	High	N	Very Low
		Current Asthma Incidence	Observational	Low	Low	Low	High	N	Very Low
		Current Asthma Incidence	Observational	Low	Low	Low	High	N	Very Low
		Ever Asthma Incidence	Observational	Low	High	Low	High	N	Very Low
		Ever Asthma Incidence	Observational	Low	Low	Low	High	N	Very Low
		Ever Asthma Incidence	Observational	Low	Low	Low	High	N	Very Low
		Allergic Rhinitis Incidence	Observational	Low	Low	Low	High	N	Very Low
		Allergic Rhinitis Incidence	Observational	Low	Low	Low	High	N	Very Low
		Allergic Rhinitis Incidence	Observational	Low	Low	Low	High	N	Very Low
Song Song, et al.	2022	Depression Risk	Interventional	Low	High	Low	Low	N	Moderate
		Negative Affect	Interventional	Low	Moderate	Low	Low	N	High
		Positive Affect	Interventional	Low	Moderate	Low	Low	N	High
Michail Georgiou, et al.	2022	Physical Activity	Interventional and Observational	Concern	High	Low	Low	N	Very Low
		Physical Activity	Interventional and Observational	Concern	High	Low	Low	N	Very Low
		Restoration	Interventional and Observational	Concern	High	Low	High	N	Very Low
		Restoration	Interventional and Observational	Concern	High	Low	Low	N	Very Low
		Restoration	Interventional and Observational	Concern	High	Low	Low	N	Very Low
		Social Interaction	Interventional and Observational	Concern	High	Low	High	N	Very Low
		Social Interaction	Interventional and Observational	Concern	Moderate	Low	High	N	Very Low
Masashi Soga, et al.	2022	Health Variables	Interventional	Low	Moderate	High	Low	N	Moderate
		Wellbeing Variables	Interventional	Low	High	High	Low	N	Low
		Beneficial for Health	Interventional	Low	Moderate	High	Low	N	Moderate
Alessandro Bianconi, et al.	2023	Cardiovascular Disease Mortality	Observational	Low	High	Low	Low	N	Very Low
		Ischemic Heart Disease Mortality	Observational	Low	High	Low	Low	N	Very Low
		Cerebrovascular Disease Mortality	Observational	Low	Low	Low	Low	N	Low
Jiang L, et al.	2023	All-site Cancer Incidence	Observational	Low	Moderate	High	Low	N	Very Low
		All-site Cancer Mortality	Observational	Low	High	High	Low	N	Very Low
		Lung Cancer Incidence	Observational	Low	Moderate	Low	Low	N	Low
		Lung Cancer Mortality	Observational	Low	High	Low	Low	N	Very Low
Fangzheng Li, et al.	2023	Parkinson Disease Incidence	Observational	Low	High	Low	High	N	Very Low
		Stroke Incidence	Observational	Low	Moderate	Low	Low	N	Low
		Cerebrovascular Disease Mortality	Observational	Low	High	Low	Low	N	Very Low
		Neurodegenerative Disease Mortality	Observational	Low	Low	Low	Low	N	Low
		Stroke Mortality	Observational	Low	Moderate	Low	Low	N	Low
Xue Wang, et al.	2023	Current Asthma Risk	Observational	Low	Moderate	Low	Low	N	Low
		Asthma Risk	Observational	Low	Low	Low	Low	N	Low
		Allergic Rhinitis Risk	Observational	Low	Low	Low	Low	N	Low
Mingcheng Tang, et al.	2023	Asthma Incidence	Observational	Low	High	Low	Low	N	Very Low
		Allergic Rhinitis Incidence	Observational	Low	Moderate	Low	High	N	Very Low
		COPD Incidence	Observational	Low	High	Low	High	N	Very Low
		COPD Mortality	Observational	Low	Low	Low	Low	N	Low
		Lung Cancer Incidence	Observational	Low	High	Low	Low	N	Very Low
		Lung Cancer Mortality	Observational	Low	High	Low	High	N	Very Low
Nv-Wei Cao, et al.	2023	Allergic Rhinitis Risk	Observational	High	Moderate	Low	Low	N	Very Low
Ziquan Liu, et al.	2023	Depression Risk	Observational	High	Moderate	Low	High	N	Very Low
		Depression Risk	Observational	High	High	Low	High	N	Very Low
		Anxiety Risk	Observational	Concern	High	Low	High	N	Very Low
Rebecca Briggs, et al.	2023	Depression Risk	Interventional	Low	Moderate	Low	Low	N	High
		Anxiety Risk	Interventional	Low	High	Low	High	N	Low
		Stress	Interventional	Low	Moderate	Low	High	N	Moderate
		Health-related Quality of Life	Interventional	Low	Moderate	Low	High	N	Moderate
		Psychosocial Well-Being	Interventional	Low	Moderate	Low	Low	N	High
Chiew Jiat Rosalind Siah, et al.	2023	Depression Risk	Interventional	Concern	Moderate	Low	Low	N	Moderate
		Anxiety Risk	Interventional	Concern	High	Low	Low	N	Low
		Systolic Blood Pressure	Interventional	Concern	Moderate	Low	Low	N	Moderate
		Diastolic Blood Pressure	Interventional	Concern	High	Low	High	N	Very Low
		Heart Rate	Interventional	Concern	Moderate	Low	High	N	Low
Yasaman Sharif, et al.	2024	Diabetes Mellitus Risk	Observational	Low	Low	Low	Low	N	Low
		Obesity Risk	Observational	Low	Moderate	Low	Low	N	Low
		Hypertension Risk	Observational	Low	High	Low	Low	N	Very Low
Yongjun Bu, et al.	2024	Systolic Blood Pressure	Observational	Concern	High	Low	Low	N	Very Low
		Systolic Blood Pressure	Observational	Concern	High	Low	Low	N	Very Low
		Hypertension Risk	Observational	Concern	High	Low	High	N	Very Low
		Hypertension Risk	Observational	Concern	High	Low	Low	N	Very Low
Muhammad Mainuddin Patwary, et al.	2024	Metabolic Syndrome Risk	Observational	Low	Low	Low	Low	N	Low
Giulia Squillacioti, et al.	2024	Asthma Incidence	Observational	Concern	Moderate	Low	High	N	Very Low
		Asthma Incidence	Observational	Concern	High	Low	High	N	Very Low
Yimin Zhang, et al.	2024	Psychiatric Disorders Risk	Observational	Concern	High	low	Low	N	Very Low
		Depression Risk	Observational	Low	High	low	Low	N	Very Low
		Anxiety Risk	Observational	Concern	Moderate	low	Low	N	Very Low
		Dementia Risk	Observational	Low	Moderate	low	Low	N	Low
		Schizophrenia Risk	Observational	Low	Moderate	low	Low	N	Low
		ADHD Risk	Observational	Low	Moderate	low	Low	N	Low
Tim Cadman, et al.	2024	Postpartum Depression Risk	Observational	Concern	Low	Low	Low	N	Very Low
		Postpartum Depression Risk	Observational	Concern	Low	Low	Low	N	Very Low
		Postpartum Depression Risk	Observational	Concern	Low	Low	Low	N	Very Low
I. Panțiru, et al.	2024	Psychosocial Well-being	Interventional and Observational	Concern	High	High	Low	N	Very Low

"Interventions/exposure indicators" column, in order to highlight the results of the blue space, BLUE was used to emphasize the exposure outcomes of the blue space. In the column of "Types of included studies", we use DARK GREEN to represent the results of single Interventional studies, LIGHTER GREEN to indicate the results of both containing interventional and observational studies, while LIGHT GREEN to represent the results of single observational studies. In the "Heterogeneity" column, to facilitate the distinction of heterogeneity, we used different shades of green for differentiation. DARK GREEN indicates high heterogeneity, LIGHTER GREEN indicates moderate heterogeneity, while LIGHT GREEN indicates low heterogeneity. Unmarked colors indicate that heterogeneity is not mentioned in the literature, and its validity deserves attention.

### 3.6 Associations between green spaces exposure and health outcomes

The final confidence rating was evaluated using a stepped upgrade/downgrade scale: intervention studies had a high initial quality rating, while all observational studies were considered low-quality evidence. The following items lead to a downgrade: *Risk of bias, Inconsistent results, Indirect evidence,* and *Imprecision.* The *Dose–response gradient* is an independent escalation criterion.

#### 3.6.1 Mortality outcome

Several systematic reviews and meta-analyses have shown a significant association between green space exposure and reduced all-cause mortality. For the general population, an increase of 0.1 unit in NDVI around residential areas is associated with a 4 to 7% reduction in all-cause mortality risk (27, 28). Among the older adult population, each 0.1 unit increase in NDVI is linked to a 1% reduction in all-cause mortality risk (29). Additionally, green space exposure may reduce disease-specific mortality rates by providing a healthier living environment. For instance, every 0.1 unit increase in NDVI is associated with a 2–3% reduction in mortality from cardiovascular disease (CVD), ischemic heart disease (IHD), and cerebrovascular disease (CBVD) (30–32). Other quantitative analyses found beneficial associations between green space and mortality rates related to neurodegenerative diseases (33) and chronic obstructive pulmonary disease (34). For the older adult, each 0.1 unit increase in NDVI corresponds to a 23 to 33% lower risk of stroke mortality (29). Two studies explored potential associations between green space exposure and mortality from cancer, with quantitative analyses suggesting potentially beneficial associations with mortality from lung cancer and prostate cancer (35, 36). These studies consistently provide evidence demonstrating the beneficial effects of green space exposure on overall health risks in the general population, particularly regarding cardiovascular diseases. However, studies on mortality risks related to other diseases currently lack quantitative data, and the evidence quality is very low, necessitating cautious interpretation of the study results.

#### 3.6.2 Neurological disorders and cognitive function

Recent studies have highlighted the association between green space exposure and neurological system diseases (NSD), which is a significant concern in public health. A meta-analysis covering 15 studies investigated the relationship between greenness exposure and NSD outcomes, including cerebrovascular diseases, stroke, and neurodegenerative diseases (33). The analysis found a significant negative correlation between greenness exposure and the risk of NSD mortality or incidence/prevalence.

Specifically, two studies observed that green space exposure could be a protective factor against dementia among various environmental exposures in residential settings (37, 38). However, a dose–response study separately examined the association between greenness and dementia. It found a slight negative correlation at moderate levels of greenness exposure but no association at high levels (39).

While evidence remains limited, factors related to climate-related exposures, including air pollution (40), short-term extreme heat (41), and climate change (42), may exacerbate symptoms of Alzheimer’s disease and related dementias (ADRD) and Parkinson’s disease (PD), and disproportionately affect them. Exposure to green spaces, vegetation, or parks may mitigate the impacts of these exposures. However, existing studies are limited and inconsistent, suggesting very low levels of evidence.

Commonly used green space metrics may not capture specific outdoor green space utilization, and less investigation into policy-related and socio-economic protective characteristics, such as economic development status and education level, remains. These overlooked features could be crucial factors influencing how green space exposure mediates neurological and cognitive function.

#### 3.6.3 Cardiovascular and metabolic diseases

The relationship between green space exposure and cardiovascular diseases and metabolic health has garnered considerable attention. Previous studies indicate that meta-analyses consistently show that green space exposure reduces mortality rates associated with ischemic heart disease and cerebrovascular diseases, though evidence regarding disease incidence is inconsistent. Research suggests that green spaces can lower the risk of cerebrovascular diseases (33), but evidence regarding the impact on cardiovascular disease risk is limited (32).

Regarding type 2 diabetes mellitus, different studies indicate that greater exposure to green spaces is associated with reduced diabetes risk (32, 43, 44), potentially linked to higher community walkability (43). Multiple systematic reviews and meta-analyses have explored the relationship between green space and metabolic health factors, including obesity (45), body mass index (BMI), hypertension (HTN) (46), blood glucose (BG), and lipid profiles (44). Studies indicate that greater exposure to green spaces is associated with lower odds of hypertension, obesity, and diabetes. Normalized Difference Vegetation Index (NDVI) in residential areas is negatively correlated with the incidence of metabolic syndrome (47).

Considering the clear evidence of green spaces and mortality from CVD, there is conflicting evidence regarding the impact of green space on mortality of cardiovascular diseases and risk. Despite heterogeneous study results and low evidence levels, it appears that residential green space exposure has a latent beneficial effect on metabolic health, warranting further prospective and mechanistic research.

#### 3.6.4 Tumors

Research on green space and cancer primarily focuses on lung cancer, while studies on other cancers (breast, prostate, and skin) suggest green spaces may be protective factors, but overall evidence is very limited due to small cohort sizes (35, 36). Additionally, green space may have different impacts on cancer mortality rates for urban and rural residents, with urban residents potentially benefiting more from green spaces (35). The quality of evidence in most current studies is rated as “very low,” indicating the need for higher-quality research to establish the exact relationship between green space and cancer. Given the unclear and highly complex etiology of cancer, along with numerous confounding factors, establishing causation is challenging; thus, future research needs to assess environmental exposure factors and investigate biological mechanisms more precisely.

#### 3.6.5 Respiratory and allergic diseases

As previously mentioned, green space exposure may serve as a protective factor against lung cancer. For other chronic non-communicable respiratory diseases, only one study categorized the impact of green space on asthma incidence and COPD incidence and mortality rates for the general population. The results indicated a significant association where an increase of 0.1 in NDVI was linked to reduced asthma incidence, lung cancer incidence, and mortality risk for chronic obstructive pulmonary disease (34). Additionally, multiple studies have explored the effects of residential green spaces, including vegetation and parks, on allergic respiratory diseases such as childhood asthma and allergic rhinitis. However, these studies have produced inconsistent results (32, 48–52). Variations in measurement methods of residential green spaces, disease diagnoses, and adjustment for confounding factors across included studies may influence the outcomes. Furthermore, seasonal changes in residential green spaces and their impact on allergens have not been fully considered. Another study involving nine European cohorts suggested an association between residential green spaces and increased childhood asthma and allergic rhinitis, emphasized that different types of green spaces, such as coniferous forests, may be associated with increased respiratory disease risks (51).

Collectively, studies examining the impact of green space exposure on respiratory health outcomes indicate inconclusive evidence regarding whether green spaces act as protective factors. The complex interactions between green spaces and respiratory system health may vary across different geographical regions and climatic conditions (53). Given the distinct mechanisms underlying chronic non-communicable respiratory diseases, respiratory infections, and allergic diseases, future research should categorically explore these diseases and consider the influence of vegetation types.

The association between residential green spaces and allergic diseases in children and adolescents is an active area of environmental health research. Simultaneously, “child-friendliness” is a focal point in landscape design studies. Certain plant species may act as allergens (54); therefore, in relevant planning and design, careful consideration should be given to the selection of vegetation that could potentially trigger allergic diseases.

#### 3.6.6 Pregnancy and neonatal outcomes

According to meta-analysis results, an increase of 0.1 unit in Normalized Difference Vegetation Index (NDVI) is associated with higher birth weight (55–59). Additionally, exposure to green spaces is linked to reduced risk of low birth weight (LBW) (55–57, 59). While the association between green space exposure and preterm birth (PTB) or small-for-gestational-age (SGA) varies across studies (32, 56–59), these studies also indicate a positive trend in reducing these risks. Some studies suggest a non-linear relationship between green space exposure and birth weight, indicating that moderate levels of green space may be more beneficial than extremely high or low levels (59). The heterogeneity of these research findings suggests the presence of other factors influencing the relationship between green space exposure and pregnancy outcomes, such as socioeconomic variables, other environmental factors, and residential conditions.

#### 3.6.7 Mental health outcomes

Residential green spaces are considered a unique and potentially modifiable exposure that can reduce physiological stress and improve mental health (60). The relationship between green spaces and mental health is a multidimensional and complex research area that has garnered increasing attention in recent years. The exact impact of green space exposure on improving mental health outcomes in adults, such as reducing depression (37, 61, 62) and anxiety symptoms, shows high heterogeneity among studies (37, 61). Although short-term exposure to natural environments exhibits significant heterogeneity, minor effects suggest a decrease in depressive mood following exposure to natural environments, whereas the increase in green spaces within residential areas alone has limited effects on enhancing positive emotions (63). However, greener residential environments correspond to higher self-rated health assessments (32). For specific populations like postpartum depression, the relationship with green spaces is less significant, whereas blue spaces may pose potential risk factors (64). Nevertheless, due to high-risk bias and low-quality studies, the credibility of these results is limited. Future research should aim to reduce biases, enhance study quality, and adhere to reporting guidelines.

Some studies further support the positive impact of green spaces on mental health. Nature-based interventions (NBIs) such as gardening, green exercise, and nature-based therapies have been effective in improving mental health outcomes for adults, including those with existing mental health issues. These interventions include promoting overall mental health through gardening activities (65–68) and alleviating depression (65). Physical activities in forests have shown improvements in depression (69–71), reduction in anxiety (69, 71), enhancement of positive emotions (69, 71), reduction of anxiety symptoms (69, 71), and have been associated with lower cortisol levels (72) and systolic blood pressure reduction (70). The most effective intervention durations range from 8 to 12 weeks, with optimal dosages varying from 20 to 90 min (69). However, interventional studies may introduce additional placebo effect, which is a significant factor limiting the credibility of the results. A policy review emphasizes the importance of creating psychologically supportive urban environments for adolescents and young adults. It suggests that while cities offer opportunities for medical, educational, and economic benefits, urban environments often pose challenges to mental health. Implementing nature-based solutions within cities through parks and urban green spaces is crucial for enhancing the mental health and well-being of urban residents (73).

### 3.7 Associations between blue exposure and health outcomes

Blue Spaces refer to all forms of natural and artificial surface water bodies, which are essential components of urban environments. There is currently only two retrieved quantitative analysis of evidence regarding the association between Blue Spaces and health outcomes. The studies found that urban Blue Spaces are positively associated with decreased obesity rates, lower all-cause mortality, overall health status, and self-reported psychological health and well-being (74). Blue Spaces facilitate physical activity and play a significant role in providing restorative environments. The impact of Blue Spaces, including coastlines, on human health in residential environments appears promising, but more evidence is still needed (75). In addition to this, some studies have shown that blue spaces help enhance health through their environmental benefits (76). Wetlands and lakes play a role in air purification, reducing air pollutants, and mitigating the urban heat island effect. Water bodies can regulate local climates, lower temperatures, and provide moisture through evaporation, helping to alleviate the adverse health effects of urban heat on residents (77). Some studies suggest that exposure to blue spaces seemed to reduce the risk of certain diseases, particularly those related to environmental pollution. For example, populations living near blue spaces generally show lower rates of cardiovascular diseases (78) compared to those residing in city centers or industrial areas.

## 4 Discussion

### 4.1 Key findings

This review encompassed a total of 47 meta-analyses. The majority of these meta-analyses were observational and evaluated green space exposure using both objective and subjective parameters, although significant variations existed between studies. Overall, exposure to green spaces showed protective effects on all-cause and cardiovascular disease mortality, overall cardiovascular disease incidence, diabetes and metabolic syndrome, low birth weight, and mental illnesses. Contact with natural environments, including gardening activities, facilitated reductions in depression, anxiety, stress, and cortisol levels. Exposure to blue spaces was positively correlated with reduced all-cause mortality, overall health status, and self-reported psychological health and well-being.

In contrast, within the included systematic reviews, evidence regarding green space exposure and disease-specific mortality, cancer, asthma, and allergic rhinitis was heterogeneous and remains unclear. AMSTAR2 assessments indicated that most included systematic reviews and meta-analyses had one or more methodological limitations, potentially introducing credibility biases into the synthesized evidence.

### 4.2 Discussion of high heterogeneity in the results

In this review, we encountered significant heterogeneity across several outcomes, as indicated by high I^2^ values and Q statistics. High levels of heterogeneity can challenge the interpretation of pooled data, as they suggest that the studies may not be directly comparable. To manage this heterogeneity, we followed the GRADE guidelines to perform a detailed heterogeneity assessment for each outcome, considering heterogeneity as a factor for downgrading the strength of evidence in our statistical analysis.

Despite these efforts, we observed that for some outcomes, particularly those with highly divergent results or even contradictory findings, it was necessary to retain all studies in the synthesis, as excluding them would risk omitting valuable data. This approach allowed us to provide a more comprehensive overview, but we acknowledge that it may have introduced further complexity into the interpretation of the results.

High heterogeneity could, in part, stem from the use of different measurement methods for the same health outcomes across studies. Variability in how outcomes are assessed—whether through different scales, questionnaires, or clinical measures—can exacerbate heterogeneity and lead to less consistent results. To address this issue in future studies, we suggest that standardization of outcome measurement tools be considered, particularly for commonly assessed health outcomes. This would enhance the comparability of results and potentially reduce heterogeneity in systematic reviews.

### 4.3 Potential mechanisms underlying green space and health

Green spaces and their effect on health outcomes involve potential mediating factors, which we categorize into three aspects for discussion.

Firstly, green spaces provide ecological services themselves (2). They are part of residential environments and influence other environmental factors such as heat exposure, air pollution levels, and noise (79), which are causally linked to various health outcomes. For instance, (1) High temperatures affect thermoregulation in humans, leading to heat-related illnesses such as heatstroke, heat fatigue, and heat cramps. Long-term exposure to high temperatures increases the risk of cardiovascular diseases (80). (2) Air pollutants like particulate matter (PM2.5 and PM10), nitrogen dioxide, sulfur dioxide, and ozone contribute to respiratory diseases such as asthma, COPD, and lung cancer (81), and are associated with increased incidence and hospitalization rates for cardiovascular diseases (82). (3) Noise can cause increased psychological stress (83, 84) and elevated risk of cardiovascular diseases (85). Environmental factors impact health in multiple ways, through direct physiological effects and by influencing behaviors and mental health. Therefore, reducing exposure to these environmental risk factors is crucial for protecting public health.

Secondly, green spaces benefit residents’ health by providing social and activity settings (86). (1) Green (and blue) spaces serve as platforms that promote physical activity and social interaction (87, 88), encouraging outdoor activities such as walking, exercising, leisure, and socializing. These activities not only promote physical health but also enhance social interactions, improving mental well-being. Exercise improves cardiorespiratory function (89), prevents and manages chronic diseases (90), enhances bone health (91), and boosts cognitive function (92); (2) Social interactions provide emotional support, reduce loneliness, and help alleviate stress and anxiety (73). Green spaces offer high-quality settings for socializing and activities, which are crucial for their impact on human health. The relationship between urban design with people behavior, and health outcomes is complex, further studies are needed to explore how different urban designs and green spaces specifically impact physical activity and health outcomes.

Additionally, based on our literature review, contact with green (and blue) spaces may have direct effects on residents’ physical health. Activities in green environments can reduce stress and anxiety, improve mood, and enhance psychological well-being (69, 71). Moreover, natural elements in green environments such as trees, water bodies, and vegetation can positively impact physiological indicators like blood pressure (70).

**Figure 3:**
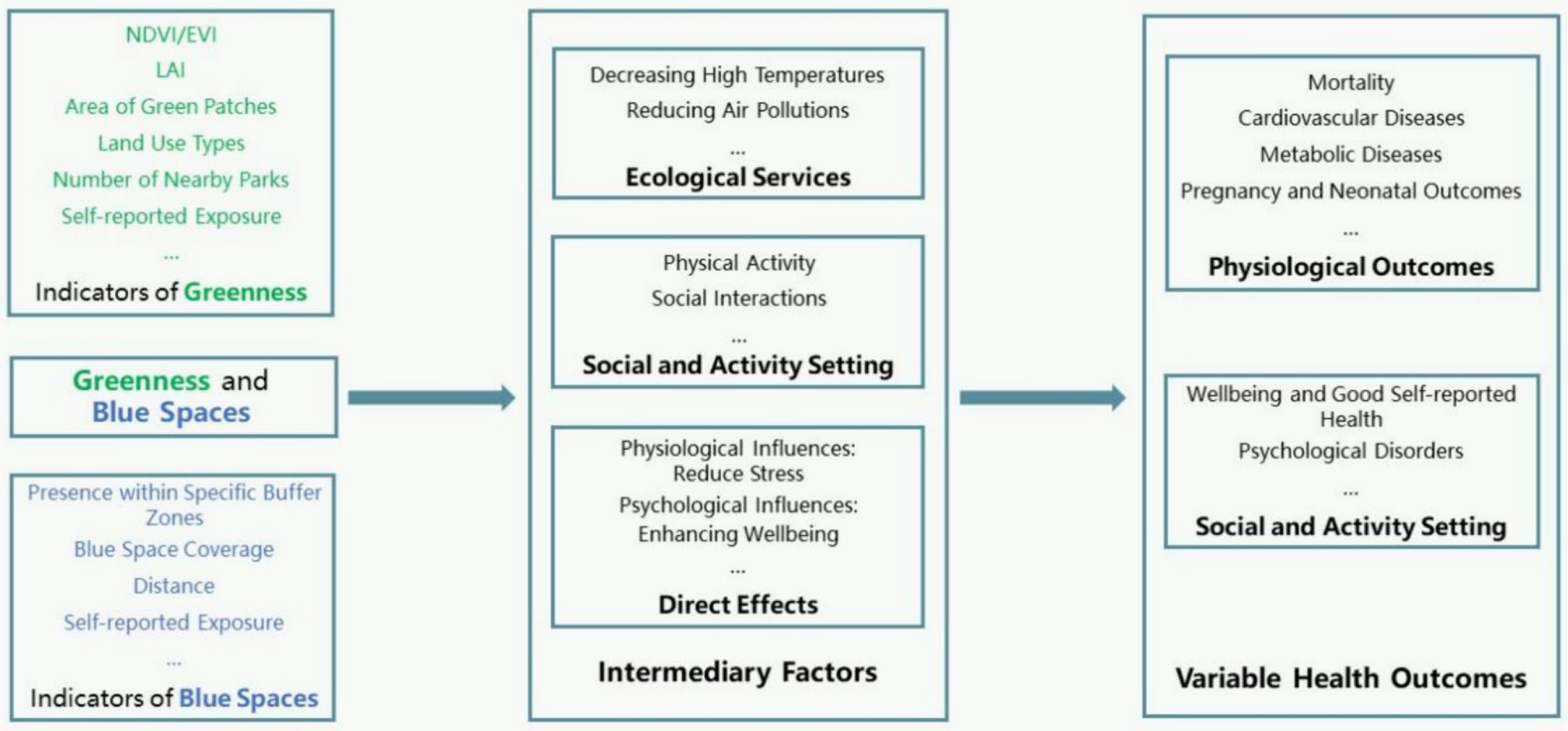
The mediation analysis model of green/blue spaces and human health.

We propose a model (Figure 3) illustrating the role of green and blue spaces in influencing human health. In this model, green spaces and blue spaces positively impact residents’ health through their different service functions, acting as “bridges” between blue/green spaces and human health. These service functions include various aspects of green spaces, such as area, quantity, distribution uniformity, accessibility, biodiversity, vegetation coverage, which reflect different service functions of green spaces. Some studies have suggested positive psychological effects of green spaces, such as biodiversity and landscape composition (93). Future research could further explore the impact and the mechanism of internal characteristics of on resident well-being, as well as regional variations in the relationship between green space service functions and resident health across different socio-economic and cultural backgrounds.

Future research should pay attention to several important issues. First, there is a risk of information loss when converting and indirectly coupling different green space indicators. Current literature reviews mostly rely on cross-sectional surveys based on NDVI/EVI indices. Therefore, future studies should not be limited to NDVI/EVI but should utilize various indicators reflecting green space. It is essential to enhance the quantitative coupling between green space indicators and ecological functions to deepen our understanding of green space ecological benefits. For example, a recent study using AI-based Google Street View assessed neighborhood features related to coronary heart disease prevalence, highlighting associations between the amount and quality of green spaces, forests, and lower CHD incidence (94). For blue spaces, further research is needed to establish standardized measures of exposure that account for aspects such as accessibility, quality, and type of water bodies (e.g., lakes, rivers, coastlines). Consistent definitions and metrics will allow for more reliable comparisons across studies and enable researchers to assess how these variables influence health outcomes. Furthermore, it is essential to explore green and blue spaces across diverse geographic and cultural contexts to verify whether the health benefits observed in one region are applicable globally.

Secondly, longitudinal studies are essential to track the long-term health effects of exposure to green and blue spaces, as cross-sectional studies cannot establish causality. These studies will identify lasting benefits and help understand how exposure across different life stages (e.g., childhood, adolescence, old age) influences health outcomes over time. Geospatial tracking can further refine this by combining environmental data with individual health trajectories. During our understanding of the interaction between health and environmental factors, it is crucial to distinguish between mediating and confounding factors. Mediating factors are part of the causal chain, acting as intermediate steps between the causal variable (green space) and the outcome variable (health). Confounding factors, on the other hand, are variables correlated with both the cause and the effect, potentially obscuring, or masking the true relationships. Factors such as gender, age, education level, regional economic status, and other environmental factors unrelated to green space should be thoroughly analyzed in layers when discussing these confounding factors to clarify causality. Many mediating factors themselves directly influence health independently of the presence of green space. It is important to further clarify the mediating effects reflected by different indicators on various health outcomes and assess their robustness to avoid biases and heterogeneity, thus guiding real-world urban design and landscape planning practices.

Thirdly, a promising direction for future research is to investigate the synergistic effects of green and blue spaces. While both types of spaces have individually been shown to benefit human health, little is known about how they may interact to enhance well-being when combined. For instance, neighborhoods that integrate both green parks and blue water features could provide cumulative health benefits beyond those offered by either type of space alone. Exploring these combined effects could inform urban planning strategies that optimize public health outcomes by integrating both green and blue spaces into urban environments.

Lastly, pure environmental ecological studies can only assess associations and mediating factors but cannot evaluate the physiological reasons behind causal relationships. Future research should integrate knowledge and techniques from multiple fields such as biology, ecology, genetics, molecular biology, and computer science under interdisciplinary backgrounds to delve into the complex mechanisms of human-environment interactions in urban living environments.

### 4.4 Strengths and limitations

Based on our comprehension, this is the second comprehensive review of evidence linking green spaces and human health, summarizing, and evaluating the evidence using systematic reviews and meta-analyses. It also represents the first systematic summary of quantitative evidence from literature that analyzes the relationship between green or blue spaces and human health in a tertiary review. This tertiary-level research surpasses primary and secondary studies in terms of evidence hierarchy. The authors thoroughly searched three international databases for relevant systematic reviews, and the two authors independently selected studies and extracted data. We strictly adhered to PRISMA guidelines and use the AMSTAR2 checklist to assess the methodological quality of the included articles. The GRADE grading system was introduced to evaluate the level of evidence in the literature. This rigorous process was able to uncover gaps and limitations in the current literature and set the stage for making recommendations to enhance future systematic reviews. Compared to the initial umbrella review in 2021, we observed progress and standardization in evidence assessment, including improved definitions of health outcomes, enhanced consideration of evidence grading, and expanded evaluation of blue spaces (7). Particularly, detailed discussions on mediating factors were added to provide guidance for future green space planning and development.

However, there are several issues to note: AMSTAR2 assessments indicated methodological limitations in most included meta-analyses, potentially undermining the credibility of synthesized evidence. Umbrella reviews can only synthesize associations between green spaces and health outcomes reported in published systematic reviews, potentially missing or underestimating associations not encompassed in these reviews. We recognize that high-quality individual studies and non-meta-analytic reviews can offer valuable insights that are not always captured in pooled analyses. Single studies with rigorous designs may highlight the nuances of green space exposure in specific populations, geographic regions, or under particular environmental conditions, which might not be well-represented in a generalized meta-analysis. Non-meta-analytic reviews often discuss theoretical frameworks or investigate specific pathways through which green space exposure affects human health, which are essential for understanding the mechanisms behind these associations. Although such studies may not provide the statistical power of meta-analyses, their qualitative contributions are indispensable for guiding future research directions and deepening our understanding of this complex relationship. The exclusion of these studies from our umbrella review represents a limitation, as it may omit important findings or perspective, such as the interaction between green space and specific socioeconomic factors (95), or the role of cultural perceptions of nature in health outcomes (96). Future researches are encouraged to integrate both meta-analytic and non-meta-analytic approaches to enrich the evidence base and provide a more holistic view of green space exposure and human health.

The green space indicators covered in this study are as previously described. Most observational studies use objective green space indicators such as NDVI as statistical metrics, which provide convenience in computing statistical measures. Linking residents’ green space exposure to NDVI using satellite imagery has become a research paradigm (97). However, this approach has limitations for many health outcomes. For example, NDVI may overlook information such as vegetation types, health conditions, and biomass within areas, and satellite-derived information is also limited by resolution (98). NDVI values are highly influenced by seasonal changes, and using NDVI values from a single time point may not accurately reflect year-round green space exposure (99). Despite the introduction of averages, this could introduce significant bias in assessing certain diseases such as cardiovascular diseases (32) and allergic diseases (32, 48–52), such as increased cardiovascular disease rates in winter and allergic diseases in spring. Other objective green space indicators, such as proximity to the nearest green space or green space ratio, also suffer from similar information losses. Although some landscape indicators that take into account heterogeneity have been proposed, they have not been widely used (100). The use of subjective assessments, such as residents’ access to green spaces, may introduce subjective biases. The quality of evidence provided by observational studies is much lower than that of intervention studies, indicating the need for more mixed-method and intervention research in the future. There remains considerable heterogeneity in health outcome assessments among studies, and pure environmental ecology alone cannot account for the sources of this heterogeneity.

We limited our review to studies published in English. English-language articles are more readily accessible and manageable for systematic review, and this allowed for a more efficient and consistent review process. This choice may introduce language bias and potentially exclude valuable international studies.

## 5 Conclusion

Overall, preliminary evidence is observed that exposure to green spaces exerts a protective influence on all-cause mortality and cardiovascular disease mortality, cardiovascular disease incidence, diabetes and metabolic syndrome, low birth weight, and mental disorders. Contact with natural environments, including gardening activities, facilitates the reduction of depression, anxiety, stress, and cortisol levels; exposure to blue spaces is positively correlated with reduced all-cause mortality, improved overall health status, and self-reported psychological well-being and happiness. However, the impact on other health outcomes is limited or uncertain. Nevertheless, these findings mainly stem from heterogeneous cross-sectional studies. Therefore, there is a necessity for longitudinal or intervention study designs to examine causality; there is a demand for more accurate quantitative assessments targeting mediating factors. For instance, dynamic assessments of green exposure using big data technologies should be implemented, considering residents’ utilization of green facilities. Studies should include more populations from low and middle-income countries. Moreover, adherence to standard guidelines in future systematic reviews is essential to bolster their methodological quality.

## Data Availability

The original contributions presented in the study are included in the article/Supplementary material, further inquiries can be directed to the corresponding author.
